# The *SLC27A1* Gene and Its Enriched PPAR Pathway Are Involved in the Regulation of Flavor Compound Hexanal Content in Chinese Native Chickens

**DOI:** 10.3390/genes13020192

**Published:** 2022-01-22

**Authors:** Yuxi Jin, Xiaoya Yuan, Wenjuan Zhao, Hua Li, Guiping Zhao, Jianfeng Liu

**Affiliations:** 1 National Engineering Laboratory for Animal Breeding and MOA Key Laboratory of Animal Genetics and Breeding, College of Animal Science and Technology, China Agricultural University, Beijing 100193, China; jinyuxi1128@163.com; 2 State Key Laboratory of Animal Nutrition, Chinese Academy of Agricultural Science, Beijing 100193, China; deletedyxy@163.com; 3 Guangdong Provincial Key Laboratory of Animal Molecular Design and Precise Breeding, Foshan University, Foshan 528225, China; zhaowj0508@163.com (W.Z.); okhuali@aliyun.com (H.L.)

**Keywords:** hexanal, RNA-seq, yellow-feathered broiler, WGCNA, *SLC27A1*

## Abstract

The role of hexanal in flavor as an indicator of the degree of oxidation of meat products is undeniable. However, the genes and pathways of hexanal formation have not been characterized in detail. In this study, we performed differential gene expression analysis and weighted gene co-expression network analysis (WGCNA) on groups of Tiannong partridge chickens with different relative hexanal content in order to find the genes involved in the formation of hexanal and the specific pathways of hexanal formation. Then we confirmed the relationship of these candidate genes with hexanal using Jingxing Yellow chicken and Wenchang chicken. In this study, WGCNA revealed a module of co-expressed genes that were highly associated with the volatile organic compound hexanal. We also compared transcriptome gene expression data of samples from chicken groups with high and low relative contents of hexanal and identified a total of 651 differentially expressed genes (DEGs). Among them, 356 genes were up regulated, and 295 genes were downregulated. The different biological functions associated with the DEGs, hub genes and hexanal were identified by functional analysis using the Kyoto Encyclopedia of Genes and Genomes (KEGG) annotations. Among all the hub genes in the significant module identified by WGCNA, more were enriched in the PPAR signaling pathway, the proteasome pathway, etc. Additionally, we found that DEGs and hub genes, including *SLC27A1*, *ACOX3*, *NR4A1*, *VEGFA*, *JUN*, *EGR1*, *CACNB1*, *GADD45A* and *DUSP1*, were co-enriched in the peroxisome proliferator-activated receptor (PPAR) signaling pathway, p53 signaling pathway and mitogen-activated protein kinases (MAPK) signaling pathway, etc. Transcriptome results of the Jingxing Yellow chicken population showed that the *SLC27A1* gene was significantly associated with hexanal and enriched in the PPAR pathway. Our study provides a comprehensive insight into the key genes related to hexanal content, and can be further explored by functional and molecular studies.

## 1. Introduction

Volatile organic compounds (VOCs) are the key constituents of meat aroma and flavor and advance the cognition of meat quality [1]. Therefore, it is important to study the changes of VOCs in meat to understand the flavor formation pathway. Hexanal, the main aroma active compound in aromatic rice milk [2] and pea milk [3], was found to be strongly correlated with lipid oxidation [4] and its relative content was increased with the storage time of the samples [5]. Chicory-fed lambs had higher hexanal content and better flavor than ryegrass-fed lambs [6]. Our previous analysis of the volatiles detected in the breast muscle of nearly 1000 different breeds of yellow-feathered broiler chickens revealed that hexanal is also the major volatile substance in chicken meat [7]. Therefore, further study of hexanal content in chicken is necessary.

Some studies have identified genes whose proteins take part in the regulation of hexanal content. For instance, after treating bananas to increase their storage time, the relative content of the compound hexanal, which contributes to their aroma, was reduced, and the activities of LOX and AAT were inhibited. Among the metabolic pathways identified, besides amino acid metabolism pathways involved in fruit ripening, lipid metabolism pathways were also affected [8]. By comparing aromatic compounds present in grapevine berry development, it was found that lipoxygenase-hydroperoxides lyase (LOX-HPL) pathway-related genes were associated with the accumulation of hexanal, while *VvLOXA* may be a crucial gene in the regulation of C6 volatiles synthesis [9]. During transport of apples, transcription factors (TFs) may lead to hexanal accumulation by regulating the expression of genes related to the LOX pathway [10]. The gas chromatography-mass spectrometry (GC-MS) technique was applied to identify hexanal as the characteristic compound in the Great River black pig dry cured ham, which was determined to be derived from the oxidation of fatty acids and the degradation of amino acids, but the specific gene involved was not identified [11]. As far as we know, a systematic and comprehensive study of the genes and pathways involved in controlling hexanal production in chicken has not been reported to date.

In this study, 398 125-day-old Tiannong partridge hens were used as experimental subjects. WGCNA was performed on relative contents of hexanal, and then 10 samples from each group with high and low relative contents of hexanal were subjected to differential gene expression analysis to identify candidate genes associated with the VOC hexanal. Further, transcriptome data from 18 samples in population of Jingxing Yellow chicken were analyzed to identify candidate genes in relation to hexanal.

## 2. Materials and Methods

### 2.1. Animals and Sampling

This study and all experimental protocols were approved by the Laboratory Animal Welfare and Animal Experimental Ethical Inspection board of Foshan University (No. 18091801). In total, 398 female Tiannong partridge chickens were obtained from Guangdong Tinoo’s Foods Group Co., Ltd. (Foshan City, China). The birds were raised in an environmentally controlled room, using commercial standard feeding. At the age of 125 days, after a 12-h overnight fast, the chickens were electrically stunned and killed by exsanguination. After slaughtering, the pectoral muscle samples were dissected from the same area of each chicken and stored at −80 °C for subsequent RNA isolation. The rest of pectoral muscle samples were stored at −80 °C for the determination of hexanal content.

### 2.2. RNA Extraction and Sequencing

Total RNA was extracted from 398 chicken pectoral muscle tissue samples using TRIzol reagent (Invitrogen, Carlsbad, CA, USA) and certain total RNA samples were selected for subsequent RNA sequencing. The RNA quality was assessed as previously described by Resnyk et al. [12]. The RNA purity was determined using a NanoPhotometer^®^-Spectrophotometer (IMPLEN Inc., West Lake Village, CA, USA) and RNA integrity and concentration were measured using the RNA Nano 6000 Assay Kit for the Bioanalyzer 2100 system (Agilent Technologies, Santa Clara, CA, USA). RNA-sequencing was performed on an Illumina NovaSeq 6000 S2 (Illumina Inc., San Diego, CA, USA) and 150-bp paired-end reads were generated [13]. We then performed quality control on the RNA data using tools Trimmomatic (v0.32) and FastQC (v0.11.7), including removal of low-quality reads (read quality < 30). Sequencing reads were aligned to the chicken reference genome [Ensembl GRCg6a (GCA_000002315.5)] by program HISAT2 [14]. Software Cufflinks (v2.0.2) was used for subsequent comparative analysis of results [15].

Transcriptome data from 18 breast muscle tissue samples of Jingxing yellow chickens (Supplementary Data 4) were uploaded in the Genome Sequence Archive [16] in BIG Data Center [17] under accession number CRA004228, CRA001908 and CRA004003 which can be publicly accessed at http://bigd.big.ac.cn/gsa (accessed on 8 March 2021). The procedure for the determination of hexanal by GC-MS analysis and the relative content of hexanal of 18 samples of Jingxing yellow chickens are shown in the literature [7].

### 2.3. Weighted Gene Correlation Network Analysis (WGCNA)

All Tiannong partridge samples were clustered to exclude any obvious outliers (cutHeight = 35,000). We then performed network construction and module detection using a step-by-step procedure. We constructed a weighted co-expression network using the thresholding power β (β = 1 to 30) to calculate adjacency between genes. Additionally, we chose the soft thresholding power (β = 8) to construct a network based on the criterion of approximate scale-free topology, using mergeCutHeight = 0.25 and minModule-Size = 30. We calculated the gene significance (GS), the correlation of the module and the gene expression profile (MM).

### 2.4. Analysis of Differentially Expressed Genes (DEGs)

From the 398 breast muscle tissues, 10 samples with high (H) and low (L) hexanal relative contents were selected to perform this analysis. The analysis of differentially expressed transcripts was performed with the edgeR package (v 4.0.2). Genes with a *p* value of less than 0.05 and absolute log2 fold change (FC) values of at least 0.585 were DEGs [18]. Hierarchical clustering analysis was performed to determine the variability and repeatability of the samples, and a volcano plot was used to visualize the overall distribution of DEGs.

### 2.5. Quantitative RT-PCR Analysis

The primers for qRT-PCR were designed by Primer premier software (v 5.0,). The forward (F) and reverse (R) primer of each gene were derived from different exons, and the size of each PCR product was about 150~250 bp. qRT-PCR was carried out in a Bio-rad CFX96 Real-Time Detection system (Bio-rad, Hercules, CA, USA) employing KAPA SYBR FAST q-PCR Kit (KAPA Biosystems, Wobrun, MA, USA) according to the manufacturer’s instructions. The 2^−(ΔΔCt)^ formula was used to quantify the relative gene expression with GAPDH as a reference gene [19].

### 2.6. Statistical Analysis

The mean and standard deviation of the relative contents of hexanal were analyzed using Microsoft Excel 2016 (Microsoft Corp., Redmond, WA, USA). The results obtained in this study were expressed as the mean ± standard deviation (SD). We used univariate analysis (*t*-test) to calculate the statistical significance (*p* value). *p* values of less than 0.05 were considered statistically significant. The biological pathways were established using the Kyoto Encyclopedia of Genes and Genomes (KEGG) database (https://www.kegg.jp/kegg/pathway.html accessed on 8 March 2021). The significance level for KEGG pathways was set to *p* < 0.05. The correlation analysis between candidate gene expression and hexanal content was performed in R statistical software (version 3.6.1) in Jingxing yellow chickens.

## 3. Results

### 3.1. Identification of Relevant Modules Associated with Hexanal

**Figure 1:**
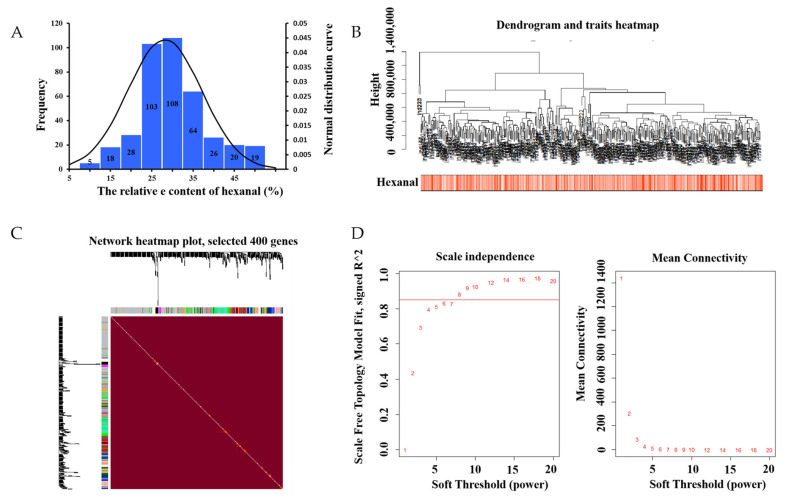
(**A**) Phenotypic distribution of 398 samples. Histogram of the frequency distribution of hexanal (%). The black line represents the normal distribution curve. (**B**) Dendrogram and traits heatmap. (**C**) Clustering dendrograms of genes, with dissimilarity based on topological overlap, together with the assigned module colors. Genes that could not be clustered into one of the two modules are labeled in gray. (**D**) Scale independence and mean connectivity.

**Figure 2:**
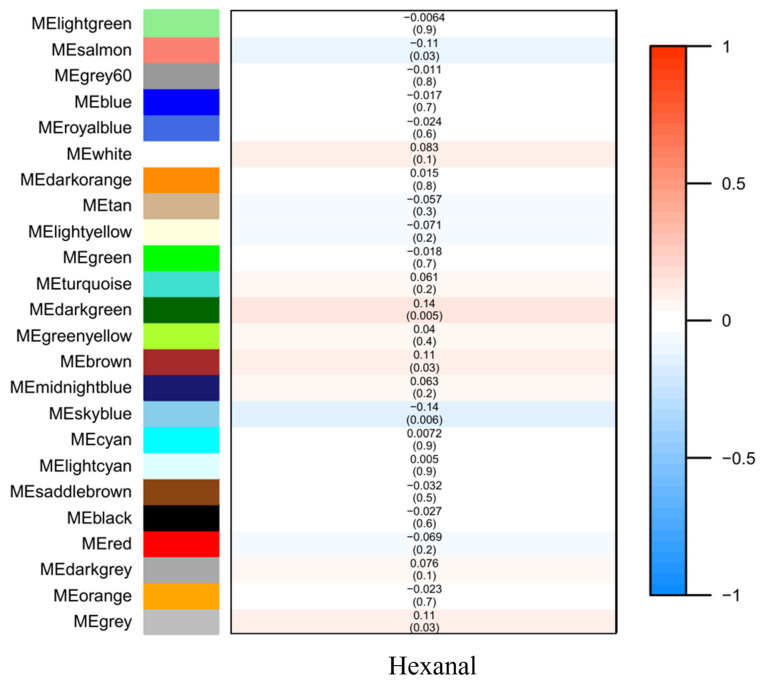
Module–trait associations. Each row corresponds to a module eigenmetabolite, column to a trait. Each cell contains the corresponding correlation and *p* value. The table is color coded by correlation according to the color legend.

The mean value of the 398 samples of hexanal relevant content was 28.14 ± 8.96% (Supplementary Data 1). In most samples, the relative content of hexanal ranged from 25–35% (Figure 1A). We then analyzed 15,093 genes to identify the related genes by WGCNA. Based on the sample clustering information, the relationship between phenotypic values and gene expression in the remaining samples after eliminating the six outliers are shown in Figure 1B. After determining a soft threshold at r2 > 0.85 (Figure 1D), 400 genes were randomly selected to show the expression patterns of genes within different modules (Figure 1C). In addition, the results showed the relationship between certain genes and hexanal content (Figure 2).

### 3.2. Further Analysis of the Significant Modules

**Figure 3:**
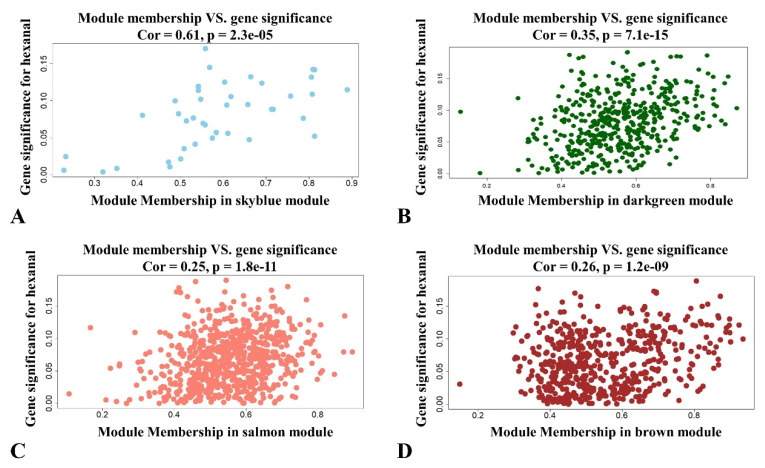
Scatterplot of module membership and gene significance showing correlation between different modules and hexanal. (**A**) Skyblue module; (**B**) Darkgreen module; (**C**) Salmon module; (**D**) Brown module.

The significance and module membership of the correlated genes were plotted for the sky blue module (r = 0.61, and *p* = 2.3 × 10^−5^), dark green module (r = 0.16, and *p* = 7.1× 10^−15^), salmon module (r = 0.25, and *p* = 1.8× 10^−11^ and brown module (r = 0.26, and *p* = 1.2× 10^−9^, again demonstrating that the genes in the modules were significantly associated with hexanal content (Figure 3). To summarize the network results, we evaluated the core genes of the relevant module from the perspective that the top network genes were ordered by the GS. A total of 438 genes were selected in four significant modules associated with hexanal (−0.20 < GS.Hexanal < 0.20, and GS.Hexanal; *p* value < 0.05, Supplementary Data 2).

### 3.3. Differentially Expressed Genes (DEGs) and Pathways Associated with Hexanal Content

**Figure 4:**
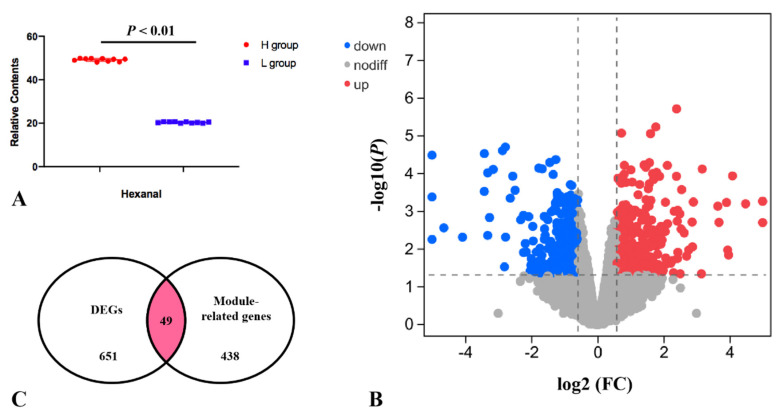
(**A**) The relative contents of hexanal for the transcriptomic profile of the H and L groups. (**B**) Volcano plot for H vs. L DEGs. (**C**) Overlap of the number of DEGs and the number of significantly related module genes in the WGCNA. K.in is described by the number of genes related to a given gene.

**Figure 5:**
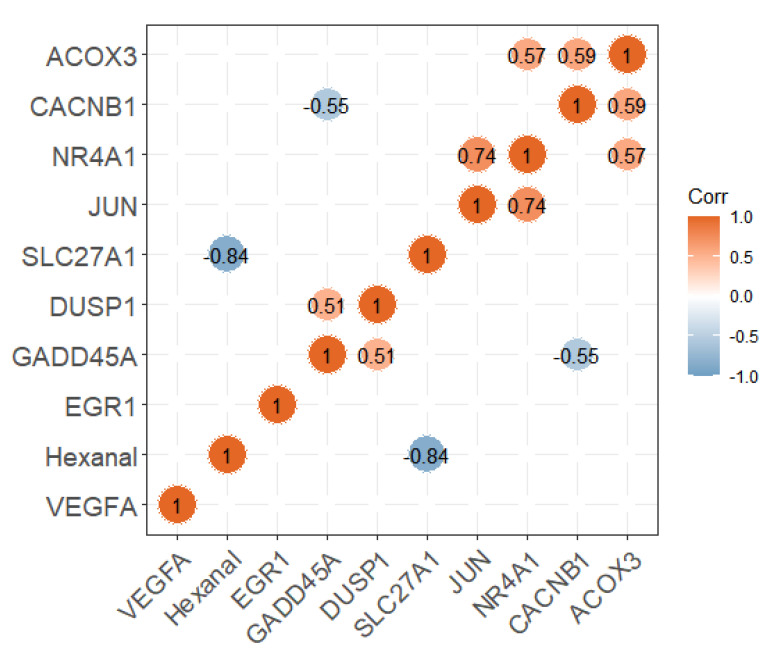
The correlation analysis between the expression of candidate genes and hexanal content.

We divided the pectoral muscle samples into two groups according to the relative content of hexanal (*p* < 0.01; Figure 4A). The DEGs between the H and L groups are shown in the volcano plot (Figure 4B). The results revealed that the expression levels of 356 and 295 genes in the H group were upregulated and downregulated, respectively, compared with the L group (Supplementary Data 3). These DEGs were significantly enriched in the PPAR signaling pathway, p53 signaling, MAPK signaling pathway etc., (Table 1). Nine of the DEGs enriched to these pathways were significantly associated with hexanal content in the WGCNA significant modules, including *SLC27A1*, *ACOX3*, *NR4A1*, *VEGFA*, *JUN*, *EGR1*, *CACNB1*, *GADD45A* and *DUSP1*. The *SLC27A1* gene was also significantly associated with hexanal content in Jingxing Yellow chickens which was enriched in the PPAR signaling pathway (Figure 5).

**Figure 6:**
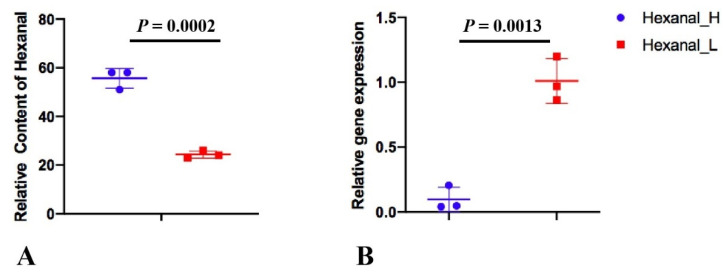
The relative content of hexanal (**A**) and the relative *SLC27A1* gene expression (**B**) in Hexanal_H and Hexanal_L groups of Jingxing Yellow chicken.

To verify the *SLC27A1* gene expression, a total of six pectoral samples (Hexanal_H ID: 492, 205 and 109; Hexanal_L ID: 406, 249 and 431) were selected from the Jingxing Yellow chicken we published [20], and we performed quantitative real-time PCR experiments (Figure 6). The primers were listed in Table 2 which were reported in this paper [21]. The gene expression pattern of quantitative real-time PCR was generally accordant with that of RNA-seq, although different in fold changes in Qingyuan partridge chicken (log2FC = −0.88), which indicated that our RNA-seq data were reliable.

## 4. Discussion

In this study, we first performed a system network analysis by transcriptome data summary statistics with hexanal content. We successfully identified four modules (brown, dark green, sky blue and salmon modules) containing 50 interconnected genes and several core genes (e.g., *SLC27A1*, *ACOX3*, and *JUN*) that are significantly associated with hexanal content. KEGG enrichment analysis revealed the relevant module enriched in the regulation of the PPAR signaling pathway, indicating a close relationship between fatty acids and hexanal content. In addition, many studies concerning the oxidation of hexanal by fatty acid, e.g., linoleic acid, have been performed [22,23]. Moreover, we also identified several interesting genes, including *SLC27A1*, which may be a promising novel candidate gene for hexanal (which will be discussed below). Thus, our findings not only confirmed that the PPAR pathway is very important to produce hexanal but also identified novel potential candidate genes for hexanal formation. Our study provides some valuable evidence to gain a better understanding of hexanal genetics and thus provide potential molecular intervention targets for hexanal.

System network analysis is a useful biological approach to identify co-expressed genes in many traits [24,25]. The major advantage of this approach is that it can find potential novel candidate modules and genes with functional features based on co-expression pattern similarity [26]. Taking this into account, we performed system network analysis by WGCNA to combine the analysis of DEGs. In this study, we identified an interesting module that showed an association with hexanal content. Pathway enrichment analysis further confirmed this specific process. Further, some studies have detected the hub genes, including the GS value [27] and intramodular connectivity [27,28].

It is worth noting that *SLC27A1* was identified as a DEG and the top network gene in the brown module. The *SLC27A1* gene was also found to be involved in the PPAR signaling pathway and downregulated in the pectoral muscle tissue of Wenchang chickens, which had a higher IMF content compared to white recessive rock chickens [21]. A significant positive correlation was also found between intramuscular fat content and hexanal in lambs given different feeding regimens [29]. Besides the *SLC27A1* gene, we also found the *ACOX3* gene, which is involved in the PPAR signaling pathway. The *ACOX3* gene, a significantly downregulated gene in Tiannong partridge chickens, is also involved in fatty acid degradation, which is in agreement with the report that its abundance was significantly lower in fast-growing chickens than in slow-growing chickens [30].

Another study showed that the expression of the *DUSP1* gene was significantly associated with high glucose levels [31]. High-fat feeding was accompanied by high glucose changes, which is also consistent with the relationship between this gene and glucolipid metabolism [32]. In our results, the relative expression level of this gene was increased in the group with high relative content of hexanal, suggesting a link among hexanal, *DUSP1* and sugar metabolism. Lipid degradation is also accompanied by changes in glucose-glutathione intermediates [33], but there is no direct evidence for a role of this gene during changes in the relative hexanal content, and additional studies are needed.

The GADD45A gene was found to contain four single nucleotide polymorphisms (SNPs) in Berkshire pigs, and all four SNPs were significantly associated with meat quality traits, such as IMF and meat color [34]. Significant differences in *GADD45A* expression were also found in the muscle transcriptional profiles of marbled and lean beef, corresponding to different lipid metabolic processes [35]. *NR4A1* was enriched in the Gene Ontology terms related to muscle development in beef cattle [36]. *VEGFA* was found to contribute to increasing adiposity in Iberian pig [37]. According to the report, *EGR1* was down-regulated via c-Fos in mature adipocytes [38]. *CACNB1* gene was reported associated with growth in chicken [39]. The *JUN* gene is a target of the heat shock transcription factor HSF1 [40]. Fewer studies on the *JUN* gene have been reported in chicken. Further studies replicating our biological experiments are needed to verify our results.

## 5. Conclusions

To understand the differences in gene expression patterns between the high hexanal group and low hexanal group in Tiannong partridge chicken, the hexanal-related genes were identified. In this study, we performed RNA-seq and characterized nearly 400 samples. Co-expression network analysis identified four modules that were associated with hexanal. The hub genes obtained using the GS value were enriched in the proteasome pathway. Moreover, besides identifying DEGs using the R software, we also identified genes related to hexanal content. Additionally, we found that DEGs and hub genes were co-enriched in the PPAR signaling pathway, Tight junction, Wnt signaling pathway, p53 signaling and MAPK signaling pathway, which included *SLC27A1*, *ACOX3*, *JUN*, *ROR1*, *GADD45A* and *DUSP1*. In Jingxing Yellow chickens, *SLC27A1* gene expression was also significantly correlated with hexanal content. These findings have revealed promising candidate genes for improvement of hexanal in further molecular studies.

## Figures and Tables

**Table 1:** The enrichment significant pathways for DEGs.

KEGG Name	Gene Name	*p* Value
PPAR signaling pathway	***ACOX3***, *ACSL1*, *ACSL4*, *APOA5*, *CD36*, *CPT1A*, *FABP4*, *PLIN2*, ***SLC27A1***	0.0004
Glycerophospholipid metabolism	*AGPAT2*, *CHKA*, *ETNK2*, *GPCPD1*, *LPCAT2*, *LPIN1*, *PCYT1B*, *PISD*, *PLA2G15*, *PLA2G4F*	0.0016
MAPK signaling pathway	*CACNA1D*, *CACNA1H*, ***CACNB1***, *CSF1*, ***DUSP1***, *DUSP8*, *EPHA2*, *FGFR1*, *FGFR2*, ***GADD45A***, *GADD45G*, ***JUN***, *MAP2K6*, *MAP4K4*, *MAPK12*, *MKNK1*, ***NR4A1***, *PLA2G4F*, ***VEGFA***	0.0026
Cytokine-cytokine receptor interaction	*BMP15*, *BMP3*, *BMPR1B*, *CCL17*, *CCL4*, *CCR5*, *CSF1*, *CXCR5*, *EDA2R*, *GDF10*, *IL12RB1*, *INHA*, *LIF*, *MSTN*	0.0164
Fatty acid biosynthesis	*ACACB*, *ACSL1*, *ACSL4*	0.0204
Fatty acid degradation	***ACOX3***, *ACSL1*, *ACSL4*, *CPT1A*	0.0276
GnRH signaling pathway	*CACNA1D*, *CAMK2A*, ***EGR1***, ***JUN***, *MAP2K6*, *MAPK12*, *PLA2G4F*	0.0280
p53 signaling pathway	*ATR*, *CCNG2*, ***GADD45A***, *GADD45G*, *GTSE1*, *SESN2*	0.0281

Note: The bold markers are genes significantly associated with hexanal content in the significant modules of WGCNA.

**Table 2:** The primers used for qRT-PCR verification.

Accession NO.	Gene Symbol	Primer Sequence	Anneaning Temperature	Product Size
NM_001039602.1	*SLC27A1*	F:TGCCTTCCGCTCTACCAC R:TCAACCCGTTTGCCCACT	59 °C	239 bp

## Data Availability

Transcriptome data were uploaded in the Genome Sequence Archive in BIG Data Center under accession number CRA004228, CRA001908 and CRA004003 which can be publicly accessed at http://bigd.big.ac.cn/gsa (accessed on 8 March 2021).

## Supplementary Materials

The following supporting information can be downloaded at: https://www.mdpi.com/article/10.3390/genes13020192/s1. Supplementary Data 1. Relative contents of hexanal in 398 samples in Tiannong Partridge chicken. Supplementary Data 2. Genes correlated with hexanal in four significant modules. Supplementary Data 3. List of differentially expressed genes. Supplementary Data 4. The expression of candidate genes and hexanal content in 18 samples of Jingxing Yellow chicken.

## Abbreviations

GS	Gene significance
MM	Module membership
WGCNA	Weight Gene Co-Expression Network Analysis
DEGs	Differential expression genes
KEGG	Kyoto Encyclopedia of Genes and Genomes
